# Kyste arachnoïdien du sinus sphénoïdal: diagnostic différentiel d’une mucocèle

**DOI:** 10.11604/pamj.2017.28.235.13958

**Published:** 2017-11-15

**Authors:** Souha Kallel, Abdel Mounem Ghorbel

**Affiliations:** 1 Service ORL et Chirurgie Cervico-faciale, CHU Habib Bourguiba, 3029 Sfax, Tunisie

**Keywords:** Kyste arachnoïdien, sinus sphénoïdal, mucocèle, Arachnoid cyst, sphenoidal sinus, mucocele

## Abstract

We here report the case of a 45 year old patient, with no previous history of head injury, presenting with isolated intermittent headache which had evolved since the young age. Clinical examination was normal. Cerebral CT scan showed osteolytic expansive hypodense lesion of the sphenoidal sinus extending inferiorly into the infratemporal fossa and superiorly into the temporal fossa (A). This mass wasn’t contrast enhanced. A mucocele was suspected. MRI was performed which showed that the mass had the same enhancement as the cerebrospinal fluid (CSF) and was in continuity with the temporal meninx, without modification in the cerebral parenchyma (B, C). The diagnosis of arachnoid cyst was retained. Given the extension of the cyst and the absence of nerve paralysis, therapeutic abstention as well as clinical and radiological monitoring were performed. Cystic lesions expanding from the sphenoid sinus are relatively rare and can cause non-specific symptoms. Even though these lesions are very rare they should be suspected. Quite often, diagnosis can be determined by CT scan data associated with MRI with diffusion-weighted sequence data, allowing the differential diagnosis of other injuries, in particular mucocele.

## Image en médecine

Il s'agissait d'une patiente âgée de 45 ans, sans antécédents de traumatisme crânien, qui a consulté pour des céphalées intermittentes isolées évoluant depuis le jeune âge. L'examen clinique était strictement normal. Le scanner cérébral a montré une lésion hypodense expansive ostéolytique du sinus sphénoïdal s'étendant vers la fosse infra-temporale en bas et la fosse temporale en haut (A). Cette masse ne prenait pas le produit de contraste. Une mucocèle a été suspectée et un complément par IRM a été réalisé. Cette dernière a montré que la masse avait le même signale que le LCR et en continuité avec la méninge temporale sans modification du parenchyme cérébral (B, C). Le diagnostic d'un kyste arachnoïdien a été retenu. Devant l'extension du kyste et l'absence de paralysie nerveuse, la décision était l'abstention thérapeutique et la surveillance clinique et radiologique. En conclision, les lésions kystiques étendues du sphénoïde sont relativement rares et peuvent provoquer des symptômes non spécifiques. Bien que ce soit une lésion très rare, le kyste arachnoïdien doit être présent à l'esprit. Le plus souvent, son diagnostic peut être déterminé à partir des données de la TDM couplée à l'IRM avec séquence de Diffusion, qui permettent le diagnostic différentiel avec les autres lésions, notamment avec une mucocèle.

**Figure 1:**
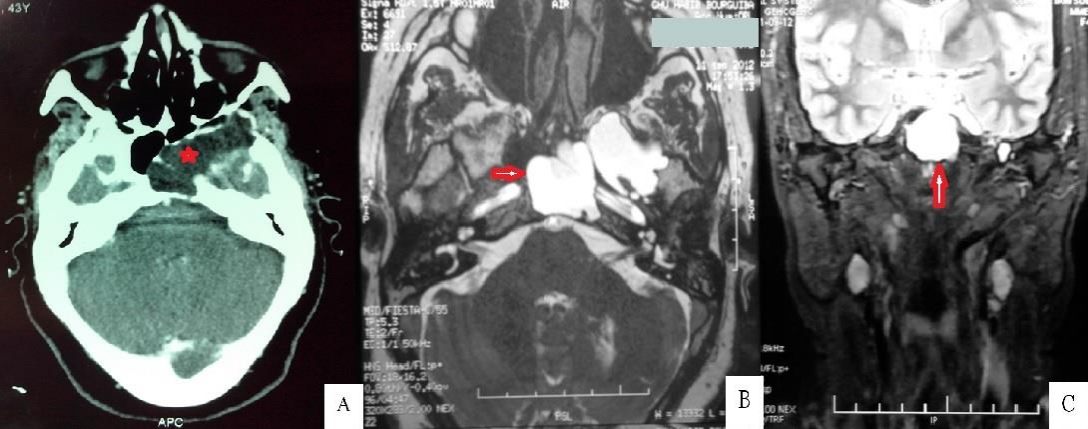
A) TDM injectée en coupe axiale: lésion hypodense expansive ostéolytique du sinus sphénoïdal s’étendant vers la fosse temporale, ne prenant pas le PDC; B) IRM coupe axiale en séquence T2: le kyste est en hypersignal franc (LCR); C) IRM coupes frontales en séquence T2 (raccordement avec la méninge temporale sans envahissement parenchymateux)

